# Effect of low-magnitude different-frequency whole-body vibration on subchondral trabecular bone microarchitecture, cartilage degradation, bone/cartilage turnover, and joint pain in rabbits with knee osteoarthritis

**DOI:** 10.1186/s12891-017-1579-0

**Published:** 2017-06-15

**Authors:** Wang Junbo, Liu Sijia, Chen Hongying, Liu Lei, Wang Pu

**Affiliations:** 1 Department of Orthopaedics, West China Hospital, Sichuan University, GuoXue Road 37, 610041 Chengdu, Sichuan People’s Republic of China; 2 Rehabilitation Medicine Center, West China Hospital, Sichuan University, Chengdu, Sichuan People’s Republic of China; 3 Key Laboratory of Rehabilitation Medicine in Sichuan, Chengdu, Sichuan People’s Republic of China; 4 Department of rehabilitation medicine, Ruijin hospital, Shanghai Jiaotong University School of Medicine, Shanghai, People’s Republic of China; 5 Department of Rehabilitation Science, Shanghai Jiaotong University School of Medicine, Shanghai, People’s Republic of China; 6 Rui Jin Rehabilitation Hospital, Shanghai, People’s Republic of China

**Keywords:** Whole-body vibration, Trabecular bone microarchitecture, Cartilage, Knee osteoarthritis

## Abstract

**Backgroud:**

Whole-body vibration(WBV) has been suggested for the prevention of subchondral bone loss of knee osteoarthritis (OA) . This study examined the effects of different frequency of whole-body vibration on subchondral trabecular bone microarchitecture, cartilage degradation and metabolism of the tibia and femoral condyle bone, and joint pain in an anterior cruciate ligament transection (ACLT)–induced knee osteoarthritisrabbit model.

**Method:**

Ninety adult rabbits were divided into six groups: all groups received unilateral ACLT; Group 1, ACLT only; Group 2, 5 Hz WBV; Group 3, 10 Hz WBV; Group 4, 20 Hz WBV; Group 5, 30 Hz WBV; and Group 6, 40 Hz WBV. Pain was tested via weight-bearing asymmetry. Subchondral trabecular bone microarchitecture was examined using in vivo micro-computed tomography. Knee joint cartilage was evaluated by gross morphology, histology, and ECM gene expression level (aggrecan and type II collagen [CTX-II]). Serum bone-specific alkaline phosphatase, N-mid OC, cartilage oligometric protein, CPII, type I collagen, PIIANP, G1/G2 aggrecan levels, and urinary CTX-II were analyzed.

**Results:**

After 8 weeks of low-magnitude WBV, the lower frequency (10 Hz and 20 Hz) WBV treatment decreased joint pain and cartilage resorption, accelerated cartilage formation, delayed cartilage degradation especially at the 20 Hz regimen. However, the higher frequencies (30 Hz and 40 Hz) had worse effects, with worse limb function and cartilage volume as well as higher histological scores and cartilage resorption. In contrast, both prevented loss of trabeculae and increased bone turnover. No significant change was observed in the 5 Hz WBV group.

**Conclusion:**

Our data demonstrate that the lower frequencies (10 Hz and 20 Hz) of low-magnitude WBV increased bone turnover, delayed cartilage degeneration, and caused a significant functional change of the OA-affected limb in ACLT-induced OA rabbit model but did not reverse OA progression after 8 weeks of treatment.

## Background

Knee osteoarthritis (KOA), the most common degenerative joint disease, is characterized by cartilage degeneration, sclerosis of the subchondral bone, and the presence of marginal osteophytes [1]. The risk factors associated with KOA include age, sex, genetics, occupation, past injury, and obesity [2]. It is estimated that >15% of the worldwide population >60 years of age is affected by KOA [3].

The optimal management of patients with KOA involves a combination of non-pharmacological and pharmacological treatments and even surgical interventions when needed [4]. In non-pharmacological interventions, regular conservative physical activity and aerobic exercise do not exacerbate but rather benefit KOA irrespective of disease severity, pain level, and functional status, which may be important alternatives for bridging the gap between disease onset and final surgical intervention [5, 6].

Whole-body vibration (WBV), which transmits a mechanical vibration to the human body using a platform with or without exercise, has grown in popularity [7]. Researchers have started to explore the possibility that WBV combined with exercise and physical therapies may offer benefits to patients with KOA, including muscle strength [8], balance control [9], and decreased self-perception of pain [9]. Additionally, previous studies found that mechanical stimulation not only increased matrix accumulation and decreased matrix metalloproteinase production in osteoarthritic chondrocytes [10], but also enhanced collagen and chondrogenic marker expression in normal cartilage [11].

Increased bone loss and porosity in the subchondral region was considered a common phenomenon in early-stage osteoarthritis (OA) [12]. WBV frequencies of 15–90 Hz also showed some positive effects on enhancing bone mineralization [13], vascularization [14], and maturation [15] during osteoporotic bone fracture healing, which may have good potential for preventing bone loss and cartilage degeneration in patients with OA. We previously demonstrated that 4-week low-magnitude 40 Hz WBV may effectively improve subchondral bone microstructure and mechanical properties of the tibia in rabbits with early KOA [16]. In contrast, other researchers suggested that 18-week low-magnitude 35 Hz WBV accelerated cartilage degeneration and caused further functional deterioration of the KOA-affected limb in a rat model [17]. WBV promoted bone formation in the OA-affected distal femur epiphysis but did not reverse OA progression.

Frequencies of 5–50 Hz have resulted in accelerations ≤ 14 g, higher frequencies (>50Hz) are likely to cause and other side effects [18, 19]. The effects of frequency are unknown but their importance has been highlighted [20]. Therefore, we wondered whether different vibration frequencies also have different biological effects on KOA and which frequency range is most effective for this indication.

Here we hypothesized that different WBV frequencies may have different effects on OA progression by delaying or accelerating OA-related cartilage deterioration and subchondral cancellous bone loss. The different biological effects of WBV on osteoarthritic cartilage, the distal femoral epiphysis, the subchondral bone plate, and limb function were compared between control and treatment groups at 5 Hz, 10 Hz, 20 Hz, 30 Hz, and 40 Hz WBV intervention. Histological, morphological, serum, and functional assessments were performed.

## Methods

### OA animal model and animal grouping

Ninety skeletally mature New Zealand white rabbits (3.5 ± 0.8 kg, 8 months of age) were purchased from the Animal Center of Sichuan University for use in this study. The animals were kept individually in cages in a room at 22 °C ± 3 °C and 55% ± 20% humidity and a 12-h light-dark cycle. Radiographs of both femorotibial joints were taken to exclude animals with joint pathology. Left knee anterior cruciate ligament transection (ACLT) surgery was performed by a skilled surgeon as previously described [21] in accordance with the rules and guidelines of the Moral Committee on Research Animal of People’s Republic. After surgery, the rabbits were allowed free cage movement for 12 weeks to develop OA. The established OA rabbits were randomly divided into six groups (15 per group): Group 1, ACLT only; Group 2, 5 Hz WBV; Group 3, 10 Hz WBV; Group 4, 20 Hz WBV; Group 5, 30 Hz WBV; and Group 6, 40 Hz WBV.

### ACLT surgery

An analgesic (buprenorphine hydrochloride 0.03 mg/kg injected intramuscularly every 6 h) was administered preoperatively and for 3 days postoperative. Antibiotics (sulfadiazine and trimethoprim 24% 15 mg/kg injected subcutaneously twice a day) were also administered preoperatively and for 2 days postoperatively. Anesthesia for the surgical procedure was maintained with a mixture of isoflurane (Technilab, Mirabel, Quebec, Canada) in oxygen. The limb was clipped and prepared for surgery in a standard manner. A medial arthrotomy was performed on the left femoropatellar joint to permit transection of the ACL. A routine skin incision closure was created. 20 weeks after surgery, tissues were harvested after taking isoflurane overdose for euthanasia.

### WBV treatment

WBV treatment was provided by a customized magnetic levitation vibration platform. We fixed five different frequencies for the vibratory analysis: F = 5 Hz, 10 Hz, 20 Hz, 30 Hz, and 40 Hz (frequency of oscillations per second, reported in Hz) with a peak-to-peak acceleration of 0.3 g (g = gravitational acceleration). An aluminum table (60 cm in diameter, 6 mm thick) was directly clamped to the top arm of the vibration platform. Each rabbit was housed individually on the table where it was free to move during the experiment. The vibration intervention consisted of 20 min/day, 5 days/week for 8 weeks, while the control group was placed on the same platform without vibration with the same regimen. At 8 weeks after treatment, we took blood via the tail vein one day before all rabbits were euthanized, then the entire OA-affected distal femur was harvested, dissected out, and processed for micro-computed tomography (micro-CT and histomorphometry. All of the operations were performed in accordance with the rules and guidelines of the Moral Committee on Research Animals of the People’s Republic.

### Pain behavior test

A pain behavior test was performed by weight-bearing asymmetry. Weight-bearing asymmetry was measured between the contralateral and ipsilateral knees using an incapacitance tester (Linton Instrumentation, Diss, UK) that could independently measure the weight-bearing ability of each leg. The measurements were repeated three times each day for three days before sacrificed, and the % weight distribution on the left hind paw was calculated by the following formula:

$$ \mathrm{L}\mathrm{W}\mathrm{D} = \mathrm{L}\mathrm{W}/\left(\mathrm{LW} + \mathrm{RW}\right) \times 100 $$

The value of the % weight distribution on the left hind paw on each measurement day was defined as the mean of nine calculations.

### Micro-CT analysis of subchondral trabecular bone microarchitecture

The distal compartments of the femoral condyles were scanned by a high-resolution cone-beam micro-CT scanner (mCT80, Scanco Medical AG, Bassersdorf, Switzerland). The X-ray source was set at 50 kV and 200 μA with an isotropic voxel size of 25 μm and a 0.5-mm-thick aluminum filter to compare total cartilage volume (CV) and subchondral bone morphology changes between groups.

The CV assessment consisted of Xie’s protocol [22]. In brief, the distal femur of the left knee was immersed in 40/60% (v/v) Hexabrix/0.15 M PBS at 37 °C for 30 min and then scanned at 10-mm resolution in the sagittal direction. After cartilage was reconstructed by a 3-D evaluation algorithm, the CV of the distal femur was evaluated.

To assess subchondral bone morphology, we selected the apex of each medial femoral condyle as the weight-bearing regions as was done previously [23]. Similarly as previously described elsewhere [24], the subchondral bone plate and trabecular bone were segmented manually a few voxels away from the endocortical boundary based on the size of the intracortical pores. According to this criterion, the endocortical boundary splits the pore in the case of large pores, whereas the pore was included in the subchondral plate region if it was less than twice the average size of the pores in that region or if the pore size was smaller than the distance from the pore to the endosteal region. Indices of cancellous bone microstructure of medial femoral condyles were determined as follows: subchondral bone plate thickness (Pt.Th, μm), subchondral plate density, bone volume fraction (BV/TV, %), trabecular number (Tb.N, mm), trabecular thickness (Tb.Th, μm) and trabecular porosity (Tb.Sp, μm). Subchondral bone plate and trabecular bone structural parameters were calculated with CTAn software according to American Society for Bone and Mineral Research guidelines and computed in a direct 3D fashion based on the marching cubes algorithm without any model assumptions for 2D analysis.

### Macroscopic and microscopic examination of the cartilage

To determine the effect of WBV on cartilage damage in OA, macroscopic and microscopic histological examinations of the cartilage were performed according to the Osteoarthritis Research Society International (OARSI) histopathology initiatives/recommendations for histological assessments of OA in rabbits [5]. After the animals were sacrificed, all of the left lateral and medial femoral condyles (LFC and MFC) could be macroscopically scored and the macroscopic grading of the lesions in the femoral condyles was as described by Pritzker et al.[25]. We used a camera (Canon EOS1100D) to obtain the digital images of the specimens with a scale marker. These photographs were used for section-cut direction to capture the most severe lesions in each compartment.

Histologic assessments were performed on the femorotibial joints of the rabbits in all six groups. Isolated cartilage from femurs and tibias were fixed in 10% neutral buffered formalin for 24 h. The tissue blocks were then decalcified with 14% ethylenediaminetetraacetic acid (Sigma) in 10 mM phosphate buffer (pH 7.4), dehydrated through graded alcohols, cleared with toluol, and embedded in paraffin. Serial sections (6 μm) were cut at a standard site centrally in the MFC and stained with hematoxylin and eosin and, Safranin O-fast green. Two histological sections from each site were evaluated using the OARSI grade and stage system [26] by two independent board-certified veterinary pathologists (Liu SJ and Chen HY) who were blinded to the treatment groups. OARSI grading system scoring was based on the most severe histological changes within each cartilage section.

### Bone/cartilage turnover analysis

Blood specimens (5 mL) were obtained via the tail vein 1 day before the animals were sacrificed for repeated measurement of serum biochemical markers. We tested the bone formation markers including serum bone-specific alkaline phosphatase (BSALP). N-mid osteocalcin (N-mid OC), bone resorption marker including C-terminal cross-linked telopeptides of type I collagen (CTX-I), cartilage formation markers including type II collagen carboxy propeptide (CP-II), and type IIA collagen N-propeptide (PIIANP), cartilage resorption marker including cartilage oligometric protein (COMP) and C-telopeptide degradation products of type II collagen (CTX-II) in the urine, and the serum G1/G2 aggrecan levels, a marker for aggrecan turnover, were quantified with commercial enzyme-linked immunosorbent assay kits (Sigma). The samples were collected and frozen in aliquots at −80 °C. All of the assays were performed according to the protocols provided by the manufacturers and measured in duplicate on the same microtiter plate.

### Statistical analysis

The statistical analysis was done using SPSS version 19.0 software for Windows (SPSS Inc) and presented using GraphPad Prism version 6.0 (GraphPad Software). All data are expressed as mean ± SD. A comparison between groups was performed by one-way analysis of variance with a post-hoc Bonferroni test after checking for normal data distribution. Biomarker data sets were examined using the Kruskal-Wallis test with Dunn’s multiple comparison test. Significant differences were determined at *P* < 0.05. All of the data analyses were conducted by a researcher (Tanke) who was blinded to the experimental groups and effect assessments.

## Results

### Functional changes of ACLT-induced OA affected limb

**Fig. 1:**
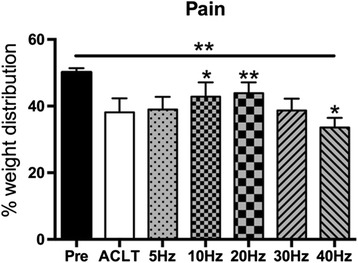
Effects of different frequency of whole-body vibration treatment on the weight distribution of the left hind paw. *P < 0.05; **P < 0.01

Figure 1 shows the % weight distribution on the left hind paw by weight-bearing asymmetry at week 8, a lower percent weight distribution on left limb means the rabbit is experiencing more pain. Compared to baseline (Pre), the % weight distribution (mean ± SD) of ACLT and WBV groups on the left hind paw were significantly reduced in all groups 8 weeks after the operation (*P* <0.01). Compared to ACLT group, there was a trend toward increasing % weight distribution on the left hind paw with increasing frequency of stimulation up to WBV groups of 10Hz (*P* <0.05) and 20 Hz (*P* <0.01), but that effect decreased at 30 Hz and 40 Hz, especially at 40Hz (*P* <0.05).

### Histological changes of articular cartilage

**Fig. 2:**
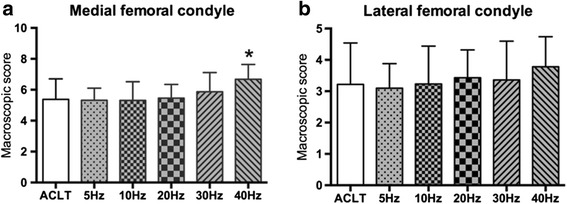
Effects of different frequencies of whole-body vibration treatment on macroscopic score of medial (**a**) and lateral (**b**) femoral condyle. *P < 0.05; **P < 0.01

Cartilage degeneration of OA was mostly developed in the femoral compartment of knee joint. Figure 2 shows representative pictures of the macroscopic histological examinations of cartilage from the ACLT and WBV groups at week 8. All specimens from the ACLT and WBV knees showed complete transection of the anterior cruciate ligament at the time of death. As the figure shows, no change was detected at frequencies of 5 Hz and 10 Hz compared to the ACLT group, but the higher frequency (≥20 Hz) WBV groups were higher than that, particularly at 40 Hz (*P* < 0.05). This change showed no significant difference between the lower frequency ( ≤ 30 Hz) WBV and ACLT groups, except the MFC of animals from the 40 Hz group showed significantly more cartilage damage than the ACLT group (*p* < 0.05). No significant difference of the macroscopic score of LFC among groups was observed. Gross analysis revealed that damage in the 40 Hz WBV groups was higher than that of the ACLT group, but no significant differences were found between the lower ones (≤30 Hz) and the ACLT group.

**Fig. 3:**
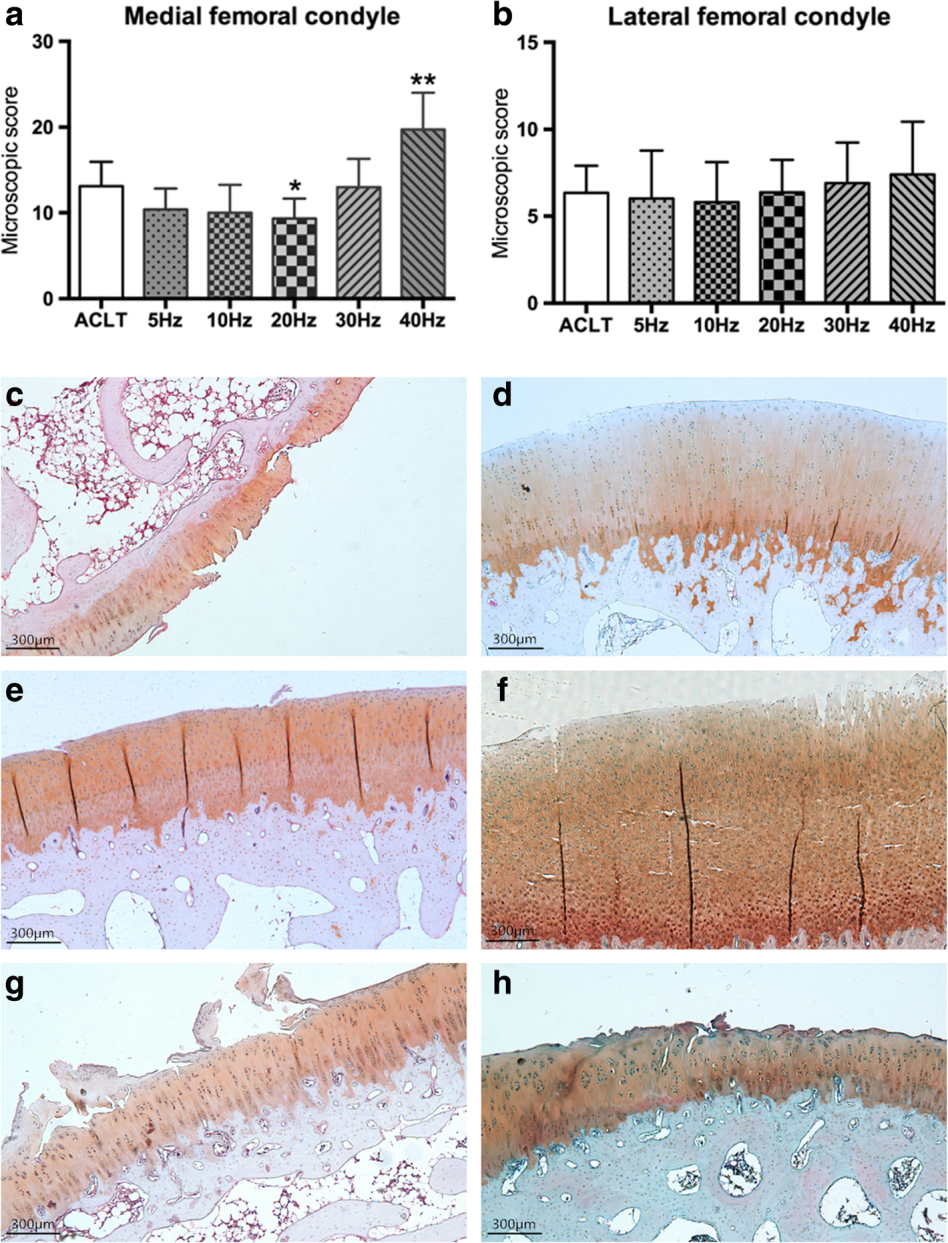
Effects of different frequencies of whole-body vibration treatment on microscopic score of medial (**a**) and lateral (**b**) femoral condyle. **a**-**f** are representative pictures from microscopic histological examinations of medial femoral condyle cartilage of the ACLT only group and the 5–40 Hz WBV groups, respectively. *P < 0.05; **P < 0.01

Figure 3 shows representative pictures from the microscopic histological examinations of cartilage from animals in the ACLT and WBV groups at week 8. We noticed that the microscopic scores of medial femoral condyles in WBV groups decrease with frequency from 5 Hz to 20 Hz below the ACLT group, especially at 20 Hz (*p* < 0.05). At higher frequencies, the microscopic scores increased and elevated up the ACLT group at 40 Hz (*p* < 0.01). Similar to macroscopic score of LFC, no differences were found between WBV and ACLT groups. These results suggest that mechanical WBV loading at 20 Hz slightly decrease the histological score and may have a protective effect on cartilage of OA rabbits, but higher frequency (40Hz) induces obvious cartilage deterioration.

### Micro-CT analysis

#### Volume change of articular cartilage

**Fig. 4:**
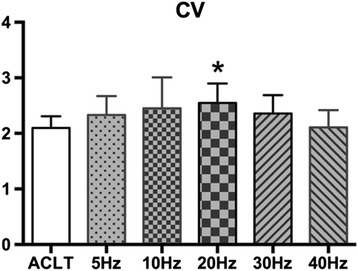
Effects of different frequencies of whole-body vibration treatment on cartilage volume (CV) of the distal femoral cartilage. *P < 0.05

The CV of the OA-affected knee joint was affected by vibration in a frequency-dependent manner. Comparing WBV groups with ACLT group, there was a trend for an increased CV with frequency up to 20 Hz (*P* < 0.05) and gradual decrease thereafter that dropped below the ACLT group at 40 Hz. These results reveal that lower frequency ( ≤ 30 Hz) WBV treatment can increase the CV of OA rabbits at week 8, whereas a higher frequency (40 Hz) had the opposite effect (Fig. 4).

#### Subchondral bone plate

**Fig. 5:**
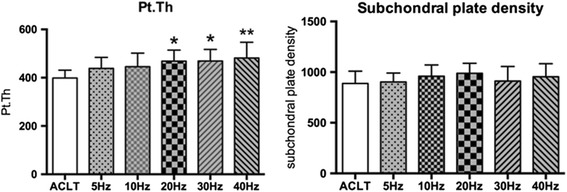
Effects of different frequency of whole-body vibration treatment on subchondral bone plate thickness (Pt.Th) and subchondral plate density of the distal femur. *P < 0.05; **P < 0.01

As Fig. 5 shows, the subchondral bone Pt.Th increased with WBV frequency, peaking at 40Hz. The Pt.Th in the 20 Hz, 30 Hz, and 40 Hz groups was significantly higher than that in the ACLT group (*P* < 0.05, *P* < 0.05, *P* < 0.01, respectively). However, no difference in the subchondral plate density among groups over WBV frequencies was observed.

#### Trabecular bone

**Fig. 6:**
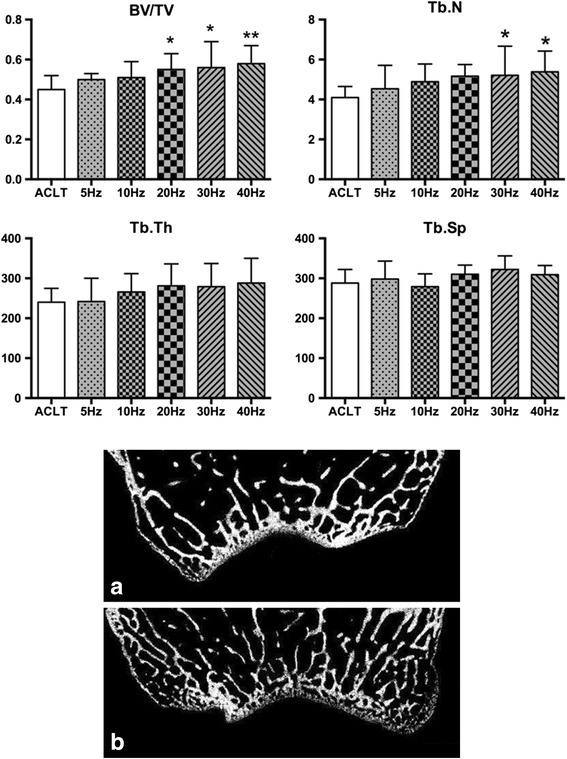
Effects of different frequencies of whole-body vibration (WBV) treatment on the microarchitecture of the trabecular bone of the distal femur. (**a**) and (**b**) are coronal micro-computed tomography slices of rabbit femoral condyles in the anterior cruciate ligament transection and WBV groups, respectively. *P < 0.05; **P < 0.01

Compared to the ACLT group in Fig. 6(A), increases in the vibration frequency resulted in a linear increase in BV/TV, Tb.N, and Tb.Th of the WBV groups. However, only the BV/TV of the 20 Hz, 30 Hz, and 40 Hz WBV groups and the Tb.N of the 30 Hz and 40 Hz groups were statistically significant (BV/TV, *P* < 0.05, *P* < 0.05, *P* < 0.01, respectively; Tb.N, *P* < 0.05, *P* < 0.05, respectively). Although trabecular bone in the 40 Hz WBV groups was a mean 48.8 μm thicker than that in the ACLT group, this difference was not statistically significant. Despite these changes in trabecular structure, no significant changes were detected in trabecular spacing (Tb.Sp) between the ACLT and WBV groups. Bone formation at the distal femoral epiphysis in the WBV group was better than that of the ACLT group, which was on a pattern of frequency, especially in the 20 Hz, 30 Hz, and 40 Hz groups.

### Evaluation of biomarkers

**Fig. 7:**
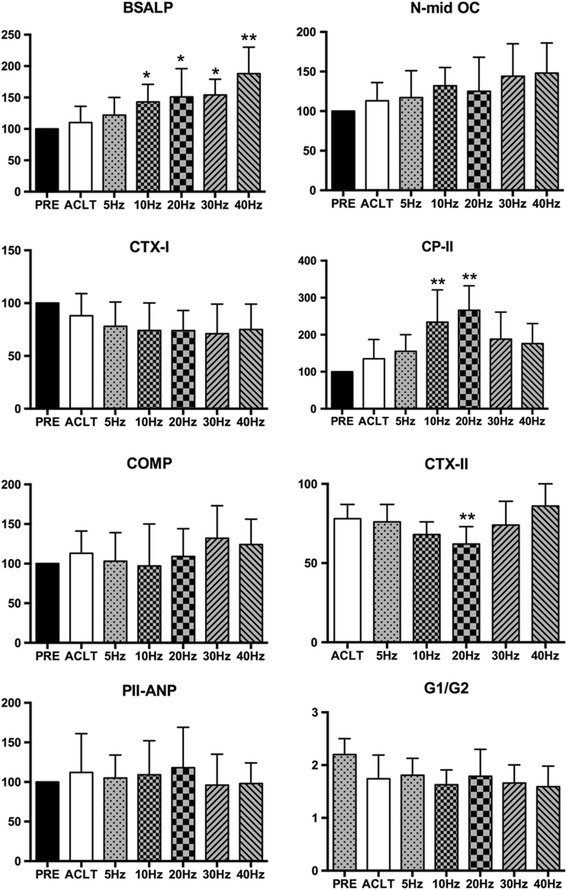
Effects of different frequencies of whole-body vibration treatment on bone and cartilage turnover. *P < 0.05; **P < 0.01

Figure 7 shows levels of the bone and cartilage turnover markers in the ACLT and WBV groups after 8 weeks of OA induction. The values of the WBV and ACLT groups were generally higher than the baseline serum BSALP and N-mid OC values, which gradually increased with vibration frequency and peaked at 40 Hz (162 and 148% of the control, respectively). Compared to the ACLT group, higher frequency (F ≥ 10 Hz) in the WBV groups appeared to have significant effects on serum BSALP, especially at 40 Hz (*P* < 0.05, *P* < 0.05, *P* < 0.05, and *P* < 0.01, respectively), but this change was not statistically significant at the serum level of N-mid OC. Compared to the ACLT group, there was a non-significant decrease in serum CTX-I in the WBV treatment groups 8 weeks after OA induction.

Regarding cartilage turnover markers, compared with baseline, there was a tendency toward increased CP-II at frequencies up to 10 Hz and 20 Hz (234 and 266% of control, respectively; *P* < 0.01) and a gradual decrease thereafter. Of note, CP-II in the 20 Hz WBV group was significantly higher than those of the 30 Hz and 40 Hz groups (*P* < 0.05, *P* < 0.01, respectively). In contrast, the CTX II level decreased as the frequency increased and peaked at 20 Hz (65% of control, *P* < 0.01), which then increased gradually and rivaled the control level (115% of control, *P* < 0.01) at 40 Hz. The CTX II level of the 40 Hz group was significantly higher than that in the 10 Hz and 20 Hz groups (*P* < 0.05 and *P* < 0.01, respectively). Although there was a similar change in the COMP level of the WBV group compared to baseline it was not statistically significant. Furthermore, we evaluated serum PIIANP and G1/G2 aggrecan as markers for CTX-II synthesis and aggrecan turnover, respectively. As Fig. 7 shows, the levels of PIIANP were not essentially different among the groups, suggesting that CTX-II synthesis can be detected by testing CPII but not PIIANP in our experimental rabbit OA model. In contrast, the levels of G1/G2 of the WBV groups and ACLT group were significantly reduced compared to baseline in the ACLT and WBV groups (*P* < 0.05), suggesting that aggrecan turnover was decreased in OA rabbits. However, compared to the ACLT group, WBV frequency had no significant effect on the reduced aggrecan turnover.

## Discussion

The main findings of this study is the differential ability of the five vibratory regimes to affect limb function, subchondral bone and cartilage structure, and formation and resorption activities in an ACLT-induced OA rabbit model. Our research revealed that lower frequency (10 Hz and 20 Hz) WBV treatment accelerated cartilage formation and decreased cartilage resorption, most significantly at the 20 Hz regimen, delayed cartilage degradation as evidenced by the lower histological score, and increased the CV after 8 weeks of WBV treatment. These findings reflected the improved functional results in the lower frequency (10 Hz and 20 Hz) WBV groups with a higher % weight distribution than the higher frequency groups (30 Hz and 40 Hz). Clearly, the higher frequencies (30 Hz and 40 Hz) were less effective than the lower frequencies. Changes were mainly evident in worse limb function and CV, higher histological scores, and accelerated cartilage resorption. In contrast, improved bone formation of the epiphysis in the distal femur was seen with increased BV/TV, Tb.N, Pt.Th, and higher serum BSALP and N-mid OC levels at the 30 Hz and 40 Hz regimens. As the previous results shows, there was non-significant change in the 5 Hz regimen in the WBV groups compared to the ACLT group.

Mechanical loading plays an important role in the onset and development of OA. Musculoskeletal tissues are highly sensitive to mechanical stimulation. However, conflicting results regarding the ability of mechanical stimulation to alter cartilage, or trabecular bone structure, in animal models and clinical studies, have recently been reported. Previous in vitro studies suggest that mid-term intermittent mechanical stimulation (cycles of 1-h sinusoidal stimulation [1 Hz] and a 4-h break; maximum compression, 2.5%) in vitro has the potential to improve the quality of cellular matrix constructs prepared from dedifferentiated osteoarthritic chondrocytes [27]. Tsuang Y-H et.al proved that mechanical stimulation (10% scaffold thickness 1-Hz amplitude lasting for 24 h) could enhance matrix protein accumulation in cultured chondrocytes [28]. However, other studies found that long-term repetitive mechanical loading (20% of the maximum isometric force of the knee joint at 0.5 Hz for 50 min/day, 3 days/week, for 4 weeks) also accelerated cartilage degeneration and increased chondrocyte death in rabbits [29]. Some other researchers have suggested that 18-week low-magnitude 35-Hz WBV accelerated cartilage degeneration and caused further functional deterioration of the KOA-affected limb in a rat model [17]. We observed similar frequency-dependent findings in OA-affected cartilage in this study. Lower frequency WBV (0.3 g, 10 Hz and 20 Hz, 20 min/day, 5 days/week for 8 weeks) treatment induced higher cartilage turnover and delayed cartilage deterioration. However, the higher frequency treatments (0.3 g, 30 Hz and 40 Hz, 20 min/day, 5 days/week for 8 weeks) led to less favorable results. One potential reason for this conclusion is the different mechanical stimulation modalities together with the different energy scales applied in these studies. Cartilage may benefit from specific mechanical stimulation modalities and a definitive energy scale. Moderate dynamic compression (10 and 20% final strain at a strain rate 100%/s, 1-h load/5-h rest cycle for 6 days) protected cartilage from mechanical injury by inhibiting the pro-catabolic response and cytokine challenge, while the strain amplitude over a particular threshold (30% final strain at a strain rate 100%/s, 1-h load/5-h rest cycle for 6 days) was detrimental to cartilage [30], indicating that lower mechanical stimuli may have protective effect on articular cartilage. Similarly in our study, 10Hz and 20Hz vibration regime are relatively moderate compared to 30Hz and 40Hz [19], which are beneficial for knee joint of osteoarthritis and might be the optimal mechanical stimulation modalities of WBV.

Subchondral and trabecular bone microarchitecture is an important determinant of bone quality [31], and many studies have reported changes in microarchitectural properties in human OA [32] and animal studies [33, 34]. In the present study, parameters of micro-CT, such as Pt.Th, BV/TV, Tb.N, and Tb.Th as well as serum markers like BSALP and N-mid-OC increased followed by no significant changes of subchondral plate density and Tb.Sp. WBV treatment prevented subchondral trabecular bone loss, preserved trabecular microarchitecture in the OA-affected distal femoral epiphysis, especially at higher frequencies (30 Hz and 40 Hz). This suggests that higher-frequency WBV treatment has the more beneficial effect on subchondral trabecular bone microarchitecture and promotes bone remodeling. The present study demonstrated the frequency dependency of the beneficial effects of WBV on the subchondral trabecular bone microarchitecture using in vivo micro-CT in an ACLT-induced rabbit model. This may result from WBV treatment (magnitude: 0.3 g; frequency: 40 Hz; time: 30 min/12 h, 5 days/week) upregulating osteoblastic activity but downregulating osteoblast-mediated osteoclastogenesis [35] and the inhibited osteoclasts formation regulated by osteocytes after low-magnitude whole-body vibration (acceleration <1 × g, where g = 9.81 m/s^2^ at 20–90 Hz) stimulation inducing enhanced bone formation and decreased bone resorption [36]. Of note, the histomorphometric parameters and bone and cartilage turnover rate increase over time was due to normal growth of the rabbit. At the early stage of OA, bone remodeling was found to increase [12] as well. However, since the time point investigated (20 weeks post-surgery) in this study was at a relatively late stage of OA, all of the rabbits were of the same age, and the OA group was compared against an age-matched control group of rabbits, the results of our study were not confounded by rabbit age.

OA is mainly characterized by painful joints. In our study, the limb function of the rabbits that received lower frequency (10 Hz and 20 Hz) WBV was higher than that of the ACLT group, especially at 20 Hz regime. However, in the higher frequency groups (30 Hz and 40 Hz), obvious joint pain was observed. Changes of bone/cartilage turnover and subchondral bone pathology observed at 8 weeks in the WBV groups coincide with decreased hind limb weight-bearing. Subchondral bone structural pathology has been shown to be associated with knee pain in OA [37]. A similar finding was observed in previous studies. WBV (2.5–5 mm displacement, 12–14 Hz, 20 min 3 times/day, 3 days/week for 8 weeks) was found to reduce pain intensity and improve quadriceps strength and dynamic balance performance in chronic KOA patients [38]. Zafar H. et al. conducted a systematic review and meta-analysis suggesting that WBV training reduces pain and improves function in individuals with KOA [39]. In our study, a moderate function change was observed in the lower frequency groups, which means that the effect of WBV in this OA model was frequency-dependent, and suggested that the lower frequency (10–20 Hz) can improve function and decrease pain.

## Conclusion

The effects of low-magnitude WBV (0.3 g, 20 min/day, 5 days/week for 8 weeks) were frequency-dependent. The lower-frequency treatments (10 Hz and 20 Hz) increased bone turnover, delayed cartilage degeneration, and caused a moderate functional change in the OA-affected limb in an ACLT-induced OA rabbit model. Further studies focusing on effects of duty cycles and long-term observations of lower frequency WBV treatments in an ACLT-induced OA model would contribute to defining the ideal time point of WBV treatment for OA, and define what effect of WBV treatment will have on normal knee joint.

## Abbreviations

ACLT: Anterior cruciate ligament transection
KOA: Knee osteoarthritis
WBV: Whole-body vibration

## References

[1] Felson DT (2000). Osteoarthritis: new insights. Part 1: the disease and its risk factors. Ann Intern Med.

[2] Blagojevic M (2010). Risk factors for onset of osteoarthritis of the knee in older adults: a systematic review and meta-analysis. Osteoarthritis Cartilage.

[3] Arden N, Nevitt MC (2006). Osteoarthritis: epidemiology. Best Pract Res Clin Rheumatol.

[4] Hochberg MC (2012). American College of Rheumatology 2012 recommendations for the use of nonpharmacologic and pharmacologic therapies in osteoarthritis of the hand, hip, and knee. Arthritis Care Res.

[5] Zhang W (2010). OARSI recommendations for the management of hip and knee osteoarthritis: part III: Changes in evidence following systematic cumulative update of research published through January 2009. Osteoarthr Cartil.

[6] Roddy E, Zhang W, Doherty M (2005). Aerobic walking or strengthening exercise for osteoarthritis of the knee? A systematic review. Ann Rheum Dis.

[7] Albasini A, Krause M, Rembitzki IV (2010). Using whole body vibration in physical therapy and sport : clinical practice and treatment exercises.

[8] Trans T (2009). Effect of whole body vibration exercise on muscle strength and proprioception in females with knee osteoarthritis. Knee.

[9] Simao AP (2012). Functional performance and inflammatory cytokines after squat exercises and whole-body vibration in elderly individuals with knee osteoarthritis. Arch Phys Med Rehabil.

[10] De Croos JN (2006). Cyclic compressive mechanical stimulation induces sequential catabolic and anabolic gene changes in chondrocytes resulting in increased extracellular matrix accumulation. Matrix Biol.

[11] Liphardt AM (2009). Vibration training intervention to maintain cartilage thickness and serum concentrations of cartilage oligometric matrix protein (COMP) during immobilization. Osteoarthr Cartil.

[12] Burr DB, Gallant MA (2012). Bone remodelling in osteoarthritis. Nat Rev Rheumatol.

[13] Leung KS (2009). Low-magnitude high-frequency vibration accelerates callus formation, mineralization, and fracture healing in rats. J Orthop Res.

[14] Cheung WH (2012). Stimulated angiogenesis for fracture healing augmented by low-magnitude, high-frequency vibration in a rat model-evaluation of pulsed-wave doppler, 3-D power Doppler ultrasonography and micro-CT microangiography. Ultrasound Med Biol.

[15] Shi HF (2010). Low-magnitude high-frequency vibration treatment augments fracture healing in ovariectomy-induced osteoporotic bone. Bone.

[16] Wang P (2014). Effects of whole body vibration on structural and functional remodeling of subchondral bones in rabbits with early osteoarthritis. Sichuan Da Xue Xue Bao Yi Xue Ban.

[17] Qin J (2014). Low magnitude high frequency vibration accelerated cartilage degeneration but improved epiphyseal bone formation in anterior cruciate ligament transect induced osteoarthritis rat model. Osteoarthr Cartil.

[18] Cheung WH (2007). High-frequency whole-body vibration improves balancing ability in elderly women. Arch Phys Med Rehabil.

[19] Rubin C (2003). Transmissibility of 15-hertz to 35-hertz vibrations to the human hip and lumbar spine: determining the physiologic feasibility of delivering low-level anabolic mechanical stimuli to skeletal regions at greatest risk of fracture because of osteoporosis. Spine (Phila Pa 1976).

[20] Torvinen S (2002). Effect of a vibration exposure on muscular performance and body balance. Randomized cross-over study. Clin Physiol Funct Imaging.

[21] Leung KS, Qin L, Cheung WH. A practical manual for musculoskeletal research. Singapore: World Scientific Publishing Company; 2008.

[22] Xie L (2012). Quantitative imaging of cartilage and bone morphology, reactive oxygen species, and vascularization in a rodent model of osteoarthritis. Arthritis Rheum.

[23] Florea C (2015). Alterations in subchondral bone plate, trabecular bone and articular cartilage properties of rabbit femoral condyles at 4 weeks after anterior cruciate ligament transection. Osteoarthr Cartil.

[24] Parfitt AM (1987). Bone histomorphometry: standardization of nomenclature, symbols, and units. Report of the ASBMR Histomorphometry Nomenclature Committee. J Bone Miner Res.

[25] Pritzker KP (2006). Osteoarthritis cartilage histopathology: grading and staging. Osteoarthr Cartil.

[26] Wang P (2014). Effects of low-level laser therapy on joint pain, synovitis, anabolic, and catabolic factors in a progressive osteoarthritis rabbit model. Lasers Med Sci.

[27] Halbwirth F (2015). Mechanostimulation changes the catabolic phenotype of human dedifferentiated osteoarthritic chondrocytes. Knee Surg Sports Traumatol Arthrosc.

[28] Tsuang YH (2008). Effect of dynamic compression on in vitro chondrocyte metabolism. Int J Artif Organs.

[29] Horisberger M (2013). Long-term repetitive mechanical loading of the knee joint by in vivo muscle stimulation accelerates cartilage degeneration and increases chondrocyte death in a rabbit model. Clin Biomech (Bristol, Avon).

[30] Li Y (2013). Moderate dynamic compression inhibits pro-catabolic response of cartilage to mechanical injury, tumor necrosis factor-alpha and interleukin-6, but accentuates degradation above a strain threshold. Osteoarthr Cartil.

[31] Dempster DW (2000). The contribution of trabecular architecture to cancellous bone quality. J Bone Miner Res.

[32] Bobinac D (2003). Changes in articular cartilage and subchondral bone histomorphometry in osteoarthritic knee joints in humans. Bone.

[33] Botter SM (2006). Quantification of subchondral bone changes in a murine osteoarthritis model using micro-CT. Biorheology.

[34] Brouwers JE (2008). Bone degeneration and recovery after early and late bisphosphonate treatment of ovariectomized wistar rats assessed by in vivo micro-computed tomography. Calcif Tissue Int.

[35] Zhou Y (2015). Whole body vibration improves osseointegration by up-regulating osteoblastic activity but down-regulating osteoblast-mediated osteoclastogenesis via ERK1/2 pathway. Bone.

[36] Lau E (2010). Effect of low-magnitude, high-frequency vibration on osteocytes in the regulation of osteoclasts. Bone.

[37] Felson DT (2001). The association of bone marrow lesions with pain in knee osteoarthritis. Ann Intern Med.

[38] Park YG (2013). Therapeutic effect of whole body vibration on chronic knee osteoarthritis. Ann Rehabil Med.

[39] Zafar H (2015). Therapeutic effects of whole-body vibration training in knee osteoarthritis: a systematic review and meta-analysis. Arch Phys Med Rehabil.

